# Sustainable Recovery of Platinum Group Metals from Spent Automotive Three-Way Catalysts through a Biogenic Thiosulfate-Copper-Ammonia System

**DOI:** 10.3390/molecules28248078

**Published:** 2023-12-14

**Authors:** Mariacristina Compagnone, José Joaquín González-Cortés, María Pilar Yeste, Domingo Cantero, Martín Ramírez

**Affiliations:** 1 Department of Chemical Engineering and Food Technologies, Wine and Agrifood Research Institute (IVAGRO), Faculty of Sciences, University of Cadiz, Puerto Real, 11510 Cadiz, Spain; mariacristina.compagnone@uca.es (M.C.); martin.ramirez@uca.es (M.R.); 2 Department of Material Science, Metallurgical Engineering and Inorganic Chemistry, Institute of Research on Electron Microscopy and Materials (IMEYMAT), Faculty of Sciences, University of Cadiz, Puerto Real, 11510 Cadiz, Spain

**Keywords:** three-way catalysts, platinum group metals, gas-lift, biogas desulfurization, bioleaching

## Abstract

This study explores an eco-friendly method for recovering platinum group metals from a synthetic automotive three-way catalyst (TWC). Bioleaching of palladium (Pd) using the thiosulfate-copper-ammonia leaching processes, with biogenic thiosulfate sourced from a bioreactor used for biogas biodesulfurization, is proposed as a sustainable alternative to conventional methods. Biogenic thiosulfate production was optimized in a gas-lift bioreactor by studying the pH (8–10) and operation modes (batch and continuous) under anoxic and microaerobic conditions for 35 d. The maximum concentration of 4.9 g S_2_O_3_^2−^ L^−1^ of biogenic thiosulfate was reached under optimal conditions (batch mode, pH = 10, and airflow rate 0.033 vvm). To optimize Pd bioleaching from a ground TWC, screening through a Plackett–Burman design determined that oxygen and temperature significantly affected the leaching yield negatively and positively, respectively. Based on these results, an optimization through an experimental design was performed, indicating the optimal conditions to be Na_2_S_2_O_3_ 1.2 M, CuSO_4_ 0.03 M, (NH_4_)_2_SO_4_ 1.5 M, Na_2_SO_3_ 0.2 M, pH 8, and 60 °C. A remarkable 96.2 and 93.2% of the total Pd was successfully extracted from the solid at 5% pulp density using both commercially available and biogenic thiosulfate, highlighting the method’s versatility for Pd bioleaching from both thiosulfate sources.

## 1. Introduction

The platinum group metals (PGMs) comprise a group of precious metals, specifically platinum (Pt), palladium (Pd), rhodium (Rh), ruthenium (Ru), iridium (Ir), and osmium (Os). These metals are highly prized for their diverse properties, which make them indispensable in numerous advanced applications across medical, electronic, and industrial domains [1].

These metals are a rarity in the Earth’s upper continental crust, with ore deposits typically containing only 3–4 g of PGMs per ton of ore. The bulk of PGM reserves can be found in South Africa (accounting for approximately 95% of global reserves) and Russia (about 2%), resulting in a significant dependence on these sources within the industry [2].

Pt and Pd, in particular, are vital components of modern industry, thanks to their unique physico-chemical properties [3]. According to Johnson Matthey’s assessment of PGM supply and demand in 2022, the global gross demand for Pt and Pd was 211.6 and 315.6 tons, respectively [4]. Consequently, the limited availability of PGMs poses a substantial challenge, especially for the European Union (EU). In response, the European Commission included PGMs in the list of critical raw materials for the EU in 2011 [5].

Over the past few decades, the demand for PGMs has seen consistent growth due to their significant industrial value, leading to increasing concerns about sustainability [6]. Producing 1 kg of PGMs from primary mineral ores requires an estimated 20 times more energy and results in higher greenhouse gas (GHG) emissions than recycling them from secondary sources [7]. Hence, there is an urgent need to transform the abundant and sometimes hazardous waste generated by PGM industries, including those in the automotive catalyst, super alloy, electronic, space industry material, biomedical equipment, and jewelry sectors, into opportunities for metal recycling.

Since 1979, the automotive industry has been the largest consumer of PGMs, with estimates suggesting that the demand for PGMs in the automotive sector in 2020 accounted for over 65% of the global demand [8]. Catalytic converters are known to contain significantly higher concentrations of PGMs than what is found in natural ores [9]. Recycling just 2 mg of spent automotive catalysts, for instance, is estimated to spare the need to mine 150 kg of PGM ores [10]. Consequently, three-way catalysts (TWCs) emerge as a particularly rich secondary source of PGMs.

Recovering PGMs from TWCs is crucial for recycling and reducing the financial and environmental impact of mining [1,11]. PGM recovery can be achieved through pyrometallurgical and hydrometallurgical methods. Pyrometallurgy involves pre-treatment steps combined with high-temperature processes that emit greenhouse gases and pollutants, making waste management challenging [7,12,13]. On the other hand, hydrometallurgy involves the use of uses corrosive chemicals, posing environmental and health risks, and requires resource-intensive chemical production [14,15]. Furthermore, proper waste treatment and disposal are crucial in both methods to minimize their environmental footprint.

Hence, ongoing efforts focus on the development of more eco-friendly and efficient methods for PGM recovery from TWCs, such as the use of green solvents [7,16]. Thiosulfate is a chemical compound that has been used in the recovery of precious metals and PGMs from various sources [16,17,18]. This anionic complexing agent forms stable complexes with metals, allowing them to be solubilized from ore or other sources, primarily through the coordination of sulfur atoms to the metal ions [19]. This process is an alternative to the more commonly used cyanide- or aqua regia-based methods for the recovery of PGMs and other precious metals and is considered to be more environmentally friendly [7,20]. Thiosulfate leaching is generally less toxic, more selective for PGMs, and can be used under milder conditions, making it a potentially safer and more environmentally friendly option [7,20]. Additionally, it can be applied to low-grade or complex ores that are challenging to process using cyanide. Thiosulfate-based processes for metal recovery can be more challenging to implement and optimize compared to cyanide-based methods [21]. Factors such as pH, temperature, and the concentration of various reagents need to be carefully controlled to ensure efficient metal recovery [17,22]. While thiosulfate-based methods have shown promise, they have not yet completely replaced cyanide-based methods in the PGM industry due to the need for further research, development, and optimization [7,23].

During biogas desulfurization, sulfur-containing compounds like hydrogen sulfide (H_2_S) are removed from biogas. This process typically involves the use of sulfur-reducing bacteria or other microorganisms that metabolize sulfur compounds [24,25]. As a result of these microbial activities, thiosulfate (S_2_O_3_^2−^) is produced as a generally considered undesirable byproduct [26,27]. Biologically produced thiosulfate has been recently used in leaching processes for the recovery of gold and silver [28,29,30]. In the study conducted by McNeice et al. [30], they reported gold extraction efficiencies spanning a range from 20 to 60%. This was achieved through the utilization of biogenic thiosulfate, following the production of approximately 400 mg L^−1^ of biogenic thiosulfate with the aid of sodium sulfide (Na_2_S) (chemical source) in conjunction with *M. sulfidovorans* (DSMZ 11578). Pourhossein and Mousavi [29] generated a biogenic thiosulfate solution at a concentration of 350 mg L^−1^, using elemental sulfur (chemical source), with which they successfully recovered 31% of gold and 40% of silver from discarded printed circuit boards with a 1% pulp density within 48 h. The same authors generated 500 mg L^−1^ of biogenic thiosulfate and improved the recovery yield by leaching 65% of gold in 36 h at a 0.5% pulp density of the same matrix [28]. Despite the good results, this approach, which reduces waste and offers an eco-friendly and potentially cost-effective method, has not been tested for PGM leaching from TWCs until now. In addition, the use of a biological bioreactor for biogas desulfurization to generate thiosulfate has also not been studied, as elemental sulfur or sulfate are the main oxidation products [31].

In this pioneering study, we present a novel and sustainable approach to recover PGMs from a palladium/alumina (Pd/Al_2_O_3_) catalyst by harnessing renewable biogenic thiosulfate derived from a bioreactor performing the biodesulfurization of biogas. This marks the first instance in which biogenic thiosulfate has been proposed as a sustainable and eco-friendly medium for PGM recovery, representing a significant advancement in the field. Our investigation encompasses the optimization of biogenic thiosulfate production within a gas-lift bioreactor, with an in-depth examination of the effects of different operation modes (batch and continuous), pH (8–10), and electron acceptors (O_2_, NO_3_^−^ and NO_2_^−^).

Additionally, to establish a comprehensive understanding of the PGM recovery process, we draw a comparative analysis between commercially available thiosulfate and biogenic thiosulfate, evaluating their respective effectiveness in recovering PGMs from the Pd/Al_2_O_3_ catalyst. This comparative assessment provides invaluable insights into the advantages and potential of biogenic thiosulfate in the context of sustainable and environmentally responsible PGM recovery.

## 2. Results and Discussion

### 2.1. Plackett–Burman Experimental Design

To explore the relationships governing metal leaching while minimizing experimental variance, we employed a synthetic Pd/Al_2_O_3_ catalyst in a Plackett–Burman design involving 12 runs and 8 factors. Utilizing glass beakers and a solution comprising Na_2_S_2_O_3_·5H_2_O, (NH_4_)_2_SO_4_, CuSO_4_·5H_2_O, and Na_2_SO_3_, tests were meticulously conducted with continuous agitation, controlled heating, and real-time monitoring.

The results obtained through the proposed Plackett–Burman (PB) experimental design for the assessment of Pd leaching percentages in various experimental conditions have yielded noteworthy insights. The initial findings reveal a distinct outcome, particularly evident in experiment number 7, which stands out as a promising outlier (Table 1). In this specific trial, 26.0% of Pd was leached by setting the following conditions: Na_2_S_2_O_3_ 1.2 M; CuSO_4_ 0.03 M; (NH_4_)_2_SO_4_ 1.5 M; Na_2_SO_3_ 0.1 M; pH 8; T (60 °C); airflow rate (0 vvm). This result contrasted with the remaining experiments where Pd recovery remained below 2.5%. This significant disparity underscores the positive outcome of experiment number 7. 

While the presence of outliers in PB experimental designs, as observed in exp. 7, can have negative consequences, such as potentially leading to misleading results and reduced precision [32], it is important to note that the overall effect of factors is typically consistent. However, the presence of outliers may introduce some level of uncertainty when determining the significance of each individual factor. The analysis of the standardized effect size of each independent variable on the leaching of Pd is shown in Figure S1. Therefore, from this first set of experiments, it was deduced that elevated concentrations of oxygen, copper, and a high pH level demonstrate an adverse effect on Pd leaching. Conversely, higher values of temperature, ammonia, thiosulfate, and sulfite exhibit a beneficial effect, promoting and enhancing the Pd leaching process.

The outcomes observed in this study are likely attributed to various underlying factors. The detrimental impact of oxygen on thiosulfate leaching of gold and nickel from different sources is well-documented [33,34,35,36]. Oxygen can have adverse effects due to its tendency to promote the oxidation of valuable minerals and metals. This undesirable oxidation can hinder the efficiency of leaching, potentially leading to reduced yields and lower leaching rates. For example, Zhang et al. [36] investigated the oxidation rate of colloidal gold in ammonia-thiosulfate systems in the absence of copper, specifically focusing on the relationship between this rate and the concentration of dissolved oxygen. Significantly, the research outcomes illuminated that an excess of oxygen (0.5 mM) exerted an adverse impact on the dissolution of gold, while copper emerged as a more efficacious oxidizing agent than oxygen for the dissolution of gold colloid in thiosulfate solutions. Consequently, minimizing oxygen exposure or carefully controlling its presence is often a crucial factor in optimizing leaching processes for enhanced metal recovery.

Sulfite and ammonia can serve as effective reducing agents, facilitating the reduction in certain metal compounds to their soluble forms [20,22,37,38,39]. This reduction reaction increases the solubility of metals and minerals, leading to improved leaching efficiency. Additionally, sulfite can help mitigate the undesirable effects of excessive oxidation by consuming oxygen or other oxidizing agents, which can further enhance the effectiveness of leaching processes. Consequently, sulfite’s role as a reducing agent and oxygen scavenger often contributes to a positive influence on the leaching of valuable metals [20]. Sulfite also acts as a stabilizing agent for thiosulfate, preventing its decomposition and thereby extending its useful lifespan in the leaching solution [37,38]. This stability ensures that thiosulfate remains available for complexation with PGM, increasing the overall leaching efficiency.

Higher concentrations of thiosulfate increase the availability of thiosulfate ions to form stable complexes with PGMs, making them more readily accessible for leaching [7,17,40]. Xu et al. [17] found that Pd extraction from decopperized anode slime was initially around 20% at a thiosulfate concentration of 0.2 M, progressively rising to nearly 40% at 0.6 M. However, it is noteworthy that the extraction efficiency began to decline as the thiosulfate concentration continued to increase. Therefore, the slight increase in thiosulfate concentration accelerates the leaching kinetics, resulting in faster PGM dissolution. A similar trend can be observed in the leaching process of other metals. Chen et al. [41] detected a noteworthy increasing trend in the extraction of gold, which elevated from around 0.8 to 35% extraction, and in the case of silver, an increase from approximately 55 to 75% extraction was demonstrated. This trend coincided with the elevation of thiosulfate concentrations ranging from 0.01 to 0.4 M. Dong et al. [42] reported that the gold leaching percentage exhibited a notable enhancement, increasing from approximately 33% at 0.05 M of thiosulfate to 83.8% when the thiosulfate concentration was raised to 0.2 M. However, the gold leaching percentage declined to 80.3% when the thiosulfate concentration reached 0.25 M, underscoring the critical importance of determining the optimal concentration range for the process. Therefore, with a surplus of thiosulfate in the leaching solution, a larger quantity of metals can be complexed and subsequently recovered, leading to higher metal recovery yields and lower leaching times [17,43,44].

In the area of PGM leaching processes related to automotive catalysts, it is widely recognized that elevated temperatures play a pivotal role in enhancing reaction kinetics. This phenomenon is evident in various approaches, encompassing both conventional methods, such as pyrometallurgical and hydrometallurgical techniques, as well as novel biological methodologies [1,7,18]. Elevated temperatures can improve the solubility of target metals, resulting in higher metal recovery efficiency. For instance, in the context of smelting processes, where temperatures can range from 1100 to 2000 °C, a remarkable recovery rate exceeding 99% for Pt, 99% for Pd, and 97% for Rh has been achieved [18]. The impact of temperature on PGM leaching is further exemplified by research on platinum leaching from catalysts in aqua regia, wherein higher temperatures were found to enhance Pt leaching [45]. Sodium cyanide, renowned for its capacity to form stable metal complexes under elevated temperature and pressure, has been a pivotal lixiviant in PGM leaching from spent automotive catalysts. An investigation into the effect of temperature and pressure on cyanide leaching of PGMs revealed temperature-dependent improvements in leaching efficiency for Pd, Pt, and Rh. Pd increased from 96% at 100 °C to 98% at 160 °C, Pt rose from 92 to 97%, and Rh increased from 89 to 93% over the same temperature range. [46]. Additionally, the role of temperature, pH, and glycine concentration in cyanide production and PGM recovery using the bacterium *C. violaceum* DSM 30,191 has been explored. This two-stage bioleaching process involved biologically producing cyanide and subsequently utilizing it for PGM recovery. The leaching efficiencies displayed a temperature-dependent trend, with recovery rates progressing from 67% at 100 °C to 80% at 150 °C, followed by a subsequent decrease. This trend aligns with prior research findings on the subject [46,47]. This phenomenon underscores the importance of an elevated temperature in achieving higher leaching efficiency, primarily attributable to the robust metallic bonds within PGM compounds.

In the study conducted by Chen et al. [16], the impact of copper concentration on gold and silver extraction within thiosulfate solutions is explored. Notably, gold extraction exhibits a remarkable upsurge from 24 to 66% for gold and from 2 to 26% for silver with a modest increment in copper concentration (0.01 to 0.1 M). Xu et al. [17] observed that palladium extraction from decopperized anode slime initially registered at approximately 28% when the copper concentration was at 0.1 M, gradually increasing to over 40% at 0.3 M. However, it is worth noting that as the copper concentration further rose, the extraction efficiency started to decrease. This observation underscores that a marginal presence of copper expedites the leaching kinetics, leading to faster dissolution of PGMs. This is probably due to the fact that high concentrations of copper may lead to competitive reactions with PGM ions [48,49]. Therefore, copper can enhance PGM solubility and facilitate their leaching by forming stable complexes with thiosulfate ions. These complexes promote PGM dissolution, leading to improved extraction rates. However, high copper concentrations can lead to the formation of excessive copper-thiosulfate complexes, which can compete with metal-thiosulfate complexes for available thiosulfate ions, reducing the efficiency of metal leaching [16,44,50,51].

### 2.2. Effect of the Main Operational Variables

**Figure 1:**
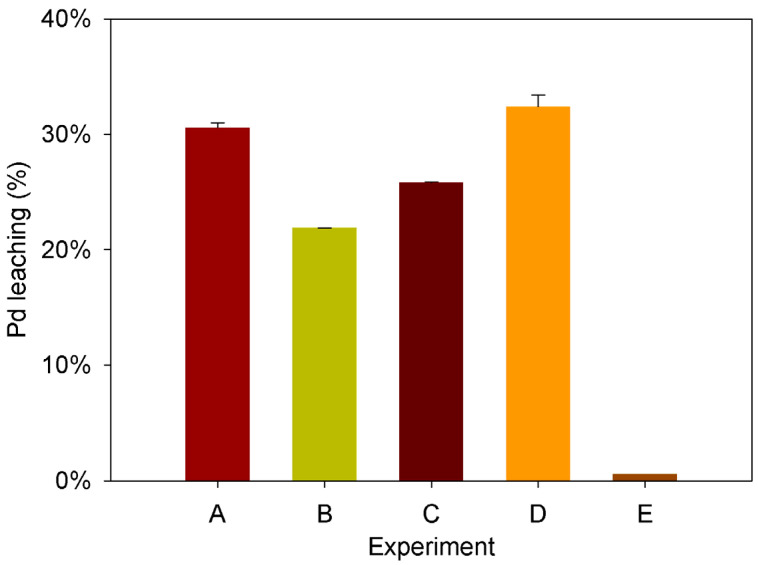
Percentage of palladium leached in the different experiments from the single-variable impact analysis. A–E represent different experimental conditions, as listed in Table 2.

To enhance Pd leaching, the levels of the chosen factors were adjusted based on the nature of the influence of each factor determined by the PB design, as illustrated in Figure S1. As a result, the concentrations of Na_2_S_2_O_3_, (NH_4_)_2_SO_4_, and Na_2_SO_3_ doubled, while the CuSO_4_ and pH values were decreased. The alterations made to the remaining factors boost metal leaching in experiments A and D (Figure 1).

In these cases, doubling the concentration of Na_2_S_2_O_3_ and Na_2_SO_3_ had a positive impact, elevating Pd leaching from the initial 26.00% (exp.7, Table 1) to 30.64 ± 0.02% and 32.41 ± 0.04%, respectively (exps. A and D, Figure 1). Experiments B and C did not largely affect the Pd leaching, indicating that doubling the NH_4_ concentration and halving the Cu concentration do not have a large impact on the process. In contrast, experiment E demonstrated that both lower and higher pH values than 8.0 significantly inhibited the leaching process to a similar extent, corroborating the pH sensitivity of this process to the pH.

These results show that, as discussed in Section 2.1, elevated thiosulfate concentrations enhance the abundance of thiosulfate ions, facilitating the formation of stable complexes with PGMs. This heightened availability renders PGMs more accessible for the leaching process [7,17,40]. Additionally, adding a small amount of sulfite to the leaching solution is a common strategy to partially reduce thiosulfate degradation in thiosulfate-based leaching processes. Thiosulfate is known to be relatively unstable and can decompose over time, which can reduce its effectiveness in recovering valuable metals, such as gold and PGMs [22,52]. Sulfite ions can act as a reducing agent, which can enhance the stability of thiosulfate and its leaching efficiency by reducing the oxidation state of metals [16,19,53].

The critical role played by the pH level in PGM leaching processes has been reported by other authors [17,54,55,56,57]. The optimal pH for leaching PGMs can vary significantly depending on the specific leaching process and conditions. Some processes favor highly acidic conditions, while others work best under neutral or slightly alkaline conditions. The choice of pH level is crucial in designing effective PGM leaching processes. Ilyas and Kim [56] document an upward trend in the extraction of Pt and Pd as the pH of the leach liquor decreases from 11.2, resulting in 96.4% Pt and 92.7% Pd extraction, to 10.4, where extraction rates reach 98.2% for Pt and 97.6% for Pd. Bax et al. [55] reported a substantial increase in the recovery of Pt and Pd at pH 1, achieving a 99.7% recovery for Pt and 96.3% for Pd. In contrast, it was observed that at pH 3, the recovery rates significantly dropped to just 11.1% for Pt and 11.8% for Pd. In view of these results, thiosulfate complexation of PGM is pH-dependent, and there is typically an optimal pH range within which the reaction is most efficient and can vary depending on the type of PGM and the associated minerals [18,49,58,59]. Deviating from this range can result in a reduced leaching rate owing to the instability of the thiosulfate complexes formed with PGM, reducing their dissolution and recovery.

Given the obtained results, it was concluded that the best conditions obtained in this study for the leaching of PGMs from TWCs were Na_2_S_2_O_3_ 1.2 M, CuSO_4_ 0.03 M, (NH_4_)_2_SO_4_ 1.5 M, Na_2_SO_3_ 0.2 M, pH 8, and 60 °C.

### 2.3. Biogenic Thiosulfate Production

**Figure 2:**
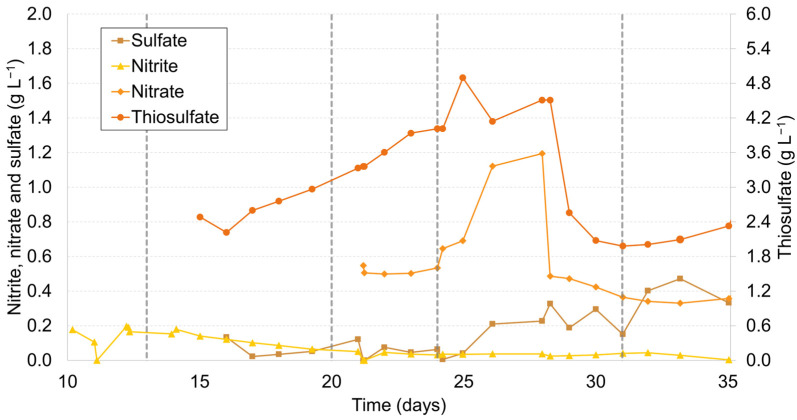
Nitrite, nitrate, sulfate, and thiosulfate concentrations measured throughout the operation of the gas-lift bioreactor.

Biological desulfurization is a well-established technique for effectively eliminating hydrogen sulfide (H_2_S) from biogas, a critical prelude to biogas valorization. However, this process typically generates byproducts, such as biogenic sulfur or thiosulfate, which traditionally have limited applications. Consequently, exploring practical uses for these biologically derived waste products is of significant interest from both environmental and economic standpoints. In this study, the production and potential maximization of biogenic thiosulfate derived from a desulfurization bioreactor were studied (Figure 2).

During Stage I, the objective was to grow the biomass and adapt it to sulfide removal from the biogas at a lower pH (8.5) to avoid excess sulfide accumulation in the liquid. From Stage II, the objective was to maximize thiosulfate production. For this purpose, the pH was raised from 8.5 to 10 and nitrite was used. After 6 days of operation, an average thiosulfate concentration of 3.02 ± 0.29 g S_2_O_3_^2−^ L^−1^ was achieved. After this stage, on day 21 (Stage III), a significant alteration was made by replacing NO_3_^−^ with NO_2_^−^ as the primary electron acceptor, with a concentration of 1 g N-NO_3_^−^ L^−1^, to assess its influence on the process. This decision was prompted by the observation that the nitrite concentration, as measured in Stage II, exhibited a slow and protracted decline, suggesting a possible scenario in which microorganisms were not efficiently utilizing nitrite. The primary difference between nitrate and nitrite is their oxidation states: nitrate is more oxidized than nitrite, usually making it a more favorable electron acceptor for microbes, as it yields more energy during its reduction [60,61]. In this particular scenario, the concentration of thiosulfate exhibited a persistent upward trend. It increased from 3.1 g S_2_O_3_^2−^ L^−1^ of biogenic thiosulfate on day 20 following the introduction of nitrite (marking the conclusion of Stage II) to 4.0 g S_2_O_3_^2−^ L^−1^ on day 24 after transitioning from nitrite to nitrate (signifying the completion of Stage III). As illustrated in Figure 2, the continuous growth in thiosulfate concentration observed in both Stage II and Stage III underscores the similar ability of nitrite and nitrate to serve as effective electron acceptors in the biogenic thiosulfate production processes.

In order to continue increasing the concentration, the liquid feed of the medium was stopped, and the bioreactor was operated in batch mode (Stage IV). This operation mode allowed the maximum thiosulfate concentration of the entire operation to be reached at 4.9 g S_2_O_3_^2−^ L^−1^ on day 25. This means that 24.5% of the total dissolved sulfide was present in the form of thiosulfate. These findings align consistently with the research conducted by Van Den Bosch [26], which demonstrated that, under alkaline pH conditions and with an H_2_S supply rate of 68 g S-H_2_S m^−3^ h^−1^, approximately 20–22% of the supplied H_2_S is transformed into thiosulfate. Notably, our current study yielded a similar conversion rate, despite employing a substantially lower supply rate of 20 g S-Na_2_S m^−3^ h^−1^, which shows that the bioprocess is reproducible and if a higher concentration of thiosulfate is to be obtained, the sulfide load must be increased. This observation underscores the consistent selectivity for biogenic thiosulfate formation, even with varying H_2_S supply rates.

While the strategy exhibited effectiveness for the initial days, with steady accumulation of thiosulfate, there was a significant subsequent decline of 60% in the thiosulfate concentration up to 2.0 g S_2_O_3_^2−^ L^−1^ on day 31. The decline in thiosulfate levels within the bioreactor serves as a clear indicator of its utilization as an energy source [62,63]. Bacteria capable of utilizing sulfide ions (HS^−^) for growth can also harness thiosulfate for their metabolic energy needs [62,64]. Transitioning the bioreactor into a batch mode prompted the accumulation of biomass, prompting these microorganisms to explore alternative energy sources in addition to sulfide ions. Consequently, thiosulfate emerged as a viable energy source under these conditions [62]. Additionally, it is important to highlight that a reduced presence of sulfide ions relative to the bacterial population correlates with diminished thiosulfate levels [65]. This correlation arises due to the chemical reactivity of sulfide, resulting in the formation of stable polysulfide compounds, which subsequently lead to thiosulfate production [65,66,67]

In an attempt to mitigate the observed decline, the introduction of oxygen into the bioreactor was ceased (Stage V). This intervention was implemented to curb the oxidation of accumulating thiosulfate to sulfate, which went from 0.006 g SO_4_^2−^ L^−1^ on day 24 to 0.333 g SO_4_^2−^ L^−1^ on day 35 as depicted in Figure 2. This correlation also gives support to the hypothesis that thiosulfate has been employed by the bacterial consortium as an energy source once the bioreactor started to operate in batch mode. Unfortunately, the outcomes of this intervention proved to be unsatisfactory, as they failed to elevate the thiosulfate concentration in the bioreactor, which reached 2.1 ± 0.17 g S_2_O_3_^2−^ L^−1^, even following an extended period of 10 days.

The observed lack of success can be ascribed to the requirement that thiosulfate production hinges on the conversion of sulfide into sulfur, leading to the formation of polysulfide compounds and, subsequently, facilitating thiosulfate production. These polysulfides are oxidized to thiosulfate only in the presence of oxygen [68] as shown in Equation (3), in Section 3.4.

The culmination of this study revealed that the highest concentration of biogenic thiosulfate, amounting to 4.9 g L^−1^, was achieved under ideal conditions. These optimal conditions encompassed the utilization of a batch operational mode, aerobic conditions, a pH level set at 10, and an airflow rate of 0.033 vvm. This outcome showcases the successful fine-tuning of the bioreactor system for enhanced biogenic thiosulfate production.

### 2.4. Comparative Analysis of PGM Recovery Using Lower Pulp Density with Biogenic and Synthetic Thiosulfate

In the context of PGM recovery from secondary sources, prior studies have indicated that higher recovery yields are achieved when employing lower percentages of solid pulps [12,14,69]. Therefore, a series of experiments was conducted by lowering the solid pulp concentration from 15 to 5%*w/v*, utilizing the previously optimized conditions (exp D, Figure 1). Additionally, these optimal conditions were tested using chemical thiosulfate and biogenic thiosulfate produced using the desulfurization bioreactor operated (Figure 2). 

**Figure 3:**
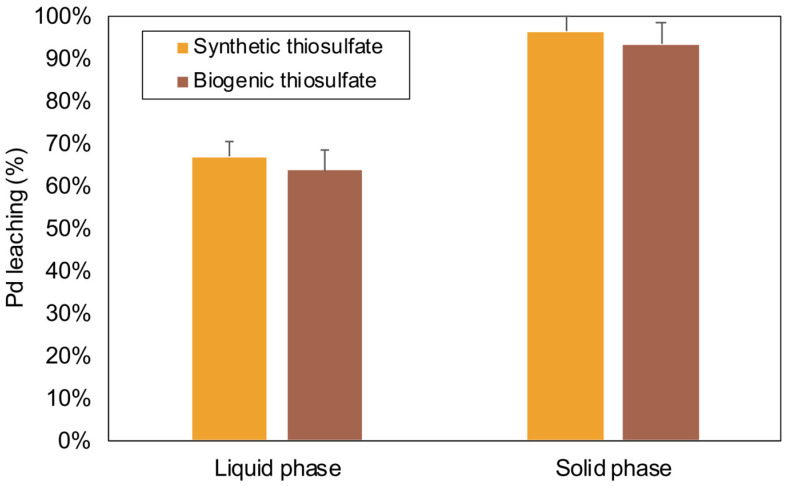
Tests at 5%*w/v* with synthetic and biogenic thiosulfate. The liquid and the solid phases were analyzed with inductively coupled plasma and X-ray fluorescence, respectively.

To comprehensively characterize these experiments under optimal conditions, both the liquid and solid samples were analyzed using inductively coupled plasma emission spectroscopy (ICP) and X-ray fluorescence (XRF). The outcomes of these analyses are visualized in Figure 3. 

The leaching of Pd has significantly improved with the use of a solid pulp concentration of 5%*w/v*, compared to the previously tested 15%*w/v*. Specifically, the Pd recovery rate detected in the liquid phase has increased from 32.41 ± 0.04% to 66.75 ± 0.04%. This noteworthy enhancement in Pd recovery highlights the substantial impact of the adjusted solid pulp concentration on the leaching process.

The effect of solid pulp concentration on the recovery of PGMs in leaching processes can vary depending on several factors, including the specific leaching method, matrix characteristics, and operational conditions [23,49,70]. Specifically, a decrease in solid pulp concentration can enhance PGM recovery from automotive catalysts in leaching and bioleaching processes [7,18,71,72]. Lowering the solid pulp concentration in the leaching system can lead to a higher liquid-to-solid ratio, which can improve mass transfer and contact between the leaching solution and the PGM-bearing material. This can enhance the dissolution and recovery of PGMs [14]. Also, high solid concentrations can lead to interference or passivation effects, where the surface of PGM particles becomes less reactive due to the accumulation of reaction products. Lower solid concentrations can mitigate these effects and allow for more efficient leaching [14,70,73].

The leaching of Pd using biologically produced thiosulfate achieved a recovery rate in the liquid phase of 63.53 ± 0.07% (Figure 3). In summary, the comparison of biogenic thiosulfate and synthetic thiosulfate described in the present study revealed nuanced differences in their impacts on the precious metal recovery process. These results underscore the promising potential of biogenic thiosulfate as an effective agent for the recovery of precious metals, emphasizing its role in sustainable and environmentally friendly metal extraction processes.

Analysis of the data presented in Figure 3 also shows that a portion of the leached Pd from the solid phase, which was successfully extracted, did not dissolve into the liquid phase. In fact, upon analyzing the solid pulp after treatment, it was determined that 96.18 ± 0.04% of Pd was extracted from the catalyst when using commercial thiosulfate and 93.22 ± 0.06% when employing biogenic thiosulfate. This occurrence is attributed to a precipitation phenomenon. Significantly, Pd displayed instability, both immediately after the leaching process and subsequent to the filtration stage (as shown in Figure S2). This instability and precipitation is likely a result of the cooling that occurs after the leaching process, causing a portion of the leachate to crystallize as the liquid and solid phases reach room temperature. [40,53]. The recovery of precious metals like Pd, Pt, and Rh from the liquid phase after a leaching process is indeed a complex process that often involves adjusting the pH and introducing a suitable precipitating agent to cause the precious metals to form insoluble compounds, which can then be separated from the liquid phase [74,75]. Additionally, as the conditions change, such as controlled cooling or evaporation of the leachate solution, precious metal compounds may precipitate out of the solution in the form of solid crystals [76]. These crystals can be further processed to obtain the pure metal forms.

In the context of our scientific discussion, it is essential to note the works of Pourhossein and Mousavi [28,29], where a biogenic thiosulfate solution generated by *Acidithiobacillus thiooxidans* was employed to recover precious metals from discarded telecommunication printed circuit boards. While Pourhossein and Mousavi [29] achieved a recovery of 31 ± 0.93% of gold and 40 ± 1.43% of silver at a 1% pulp density within a 48 h timeframe, the results were notably improved in their subsequent study [28], where the recovery rate increased to 65 ± 0.78% for gold and achieved this within 36 h at a reduced pulp density of 0.5%*w/v*. It is important to compare these outcomes with more conventional methods, such as those employed by Huang et al. [77] that used commercial thiosulfate to recover 94.5% of gold.

## 3. Materials and Methods

### 3.1. Synthetic Catalyst (Pd/Al_2_O_3_)

For the metal bioleaching tests, a synthetic catalyst Pd/Al_2_O_3_, with the Pd supported on alpha-alumina. The catalyst was prepared by Incipient wetness impregnation using a 0.5M Pd(NO_3_)_2_ solution. Three impregnation cycles were carried out to obtain 0.8% weight of Pd. Finally, the catalyst was calcined in a muffle furnace at 500 °C for one hour with a heating ramp of 5 °C min^−^^1^ to decompose the palladium precursor to palladium oxide. To obtain the Pd in the metallic state, the catalyst was reduced with a flow of 100 mL min^−^^1^ of 5%H_2_/Ar at 500 °C for 1 h (heating ramp of 5 °C min^−^^1^).

### 3.2. Screening through a Plackett–Burman Design

To explore the relationship of the independent variables (factors) and to minimize the variance of the estimates of these relationships with a limited number of experiments a PB design with 12 runs, 8 factors, and 3 degrees of freedom was conducted.

The leaching tests were carried out in glass beakers in which 15%*w/v* of solid samples were placed with a liquid solution containing Na_2_S_2_O_3_·5H_2_O, (NH_4_)_2_SO_4_, CuSO_4_·5H_2_O, and Na_2_SO_3_ at the concentrations indicated in Table 1. The tests were conducted with continuous agitation at a fixed speed of 300 rpm, and, when applicable, simultaneous heating was applied to reach 60 °C using a magnetic stirrer. LabVIEW platform (National Instruments^TM^, Austin, TX, USA) with cDAQ Chassis and three modules: a current input module (NI-9209), voltage output module (NI-9264), and digital I/O module (NI-9184) were used for monitoring the tests. pH was adjusted by the addition of 1M NaOH solution, and the value was kept steady by readjustment every 30 min. When the reaction was completed, the solutions were centrifuged at 1610× *g* for 10 min to separate the liquid phases from the solid phases, which were dried in an oven at 80 °C for 24 h.

### 3.3. Effect of the Main Operational Variables

Additional experiments were designed based on the nature of the influence (positive or negative) of the different variables on the percentage of Pd leaching obtained in PB. Thus, the value of the positively affecting variables was increased by 100% and the value of the negatively affecting variables was reduced by 50%, as shown in Table 2.

The temperature was maintained constant at 60 °C and no O_2_ was added, considering the detrimental effect of changing these values. The leaching tests were conducted using the identical procedure employed in the PB design experiments.

### 3.4. Biogenic Thiosulfate Production

**Figure 4:**
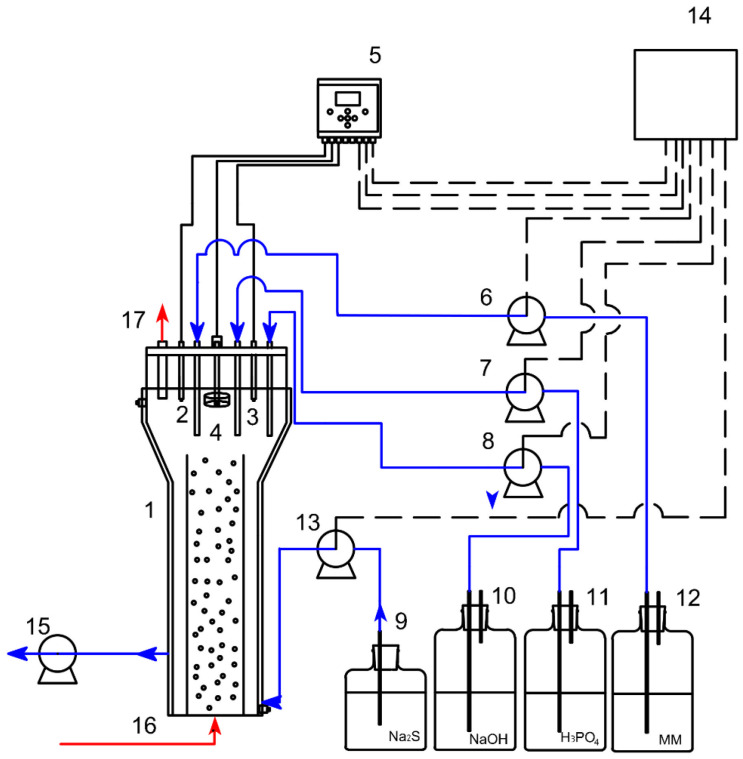
Inner loop jacketed gas-lift bioreactor scheme. 1: Gas-lift bioreactor; 2: pH probe; 3: ORP probe; 4: sensor level; 5: Multimeter; 6: Analog peristaltic pump; 7: H_3_PO_4_ peristaltic pump; 8: NaOH peristaltic pump; 9: Na_2_S container; 10: NaOH container; 11: H_3_PO_4_ container; 12: Mineral medium container; 13: Na_2_S peristaltic pump; 14: PC and control system; 15: Discharge peristaltic pump; 16: Gas flow inlet; 17: Gas flow outlet. Red arrows stand for gaseous streams while blue arrows represent liquid streams.

Thiosulfate production was studied in an inner loop jacketed gas-lift bioreactor (Applikon Biotechnology BV, Delft, The Netherlands) with a working volume of 3L (Figure 4).

The medium used had the following composition (g/L): KH_2_PO_4_ (4); NH_4_Cl (2); MgSO_4_·7H_2_O (1.6) and 0.8 mL L^−^^1^ of solution SL-4 × 5, whose composition was reported by [78]. The volume was kept constant by a sensor level (Vertical Float Switch, Cynergy3, Wimborne, UK) connected to a discharge peristaltic pump. The oxidation−reduction potential (ORP) and pH were measured with a multiparametric analyzer (Crison Multimeter 44, Hach Lange S.L.U, Barcelona, Spain). The pH was controlled by the addition of H_3_PO_4_ (2 N) and NaOH (2 N). The bioreactor was fed with Na_2_S·3H_2_O to maintain a constant inlet load (IL) of 20 g S-Na_2_S m^−3^ h^−1^. The airflow rate was maintained at 0.033 vvm throughout the entire operation. This specific airflow rate was selected due to the fact that elemental sulfur is an intermediate byproduct of the sulfide-to-sulfate oxidation process catalyzed by sulfur-oxidizing bacteria (SOB) [66]. Elemental sulfur becomes the primary product when oxygen levels are limited (Equation (1)).

HS^−^ + 0.5 O_2_ ↔ S^0^ + OH^−^ (1)

Consequently, lower concentrations of dissolved oxygen were deliberately maintained to promote biogenic sulfur production. This approach allowed the sulfide to react chemically with sulfur, resulting in the formation of stable polysulfide compounds (Equation (2)) [67].

HS^−^ + (x − 1) S^0^ ↔ Sx^2−^ + H^+^; x ≤ 6 (2)

Under alkaline pH conditions and in the presence of oxygen, these polysulfide compounds are rapidly oxidized to thiosulfate (Equation (3)) [68].

Sx^2−^ + O_2_ + 5 H_2_O ↔ 0.5 S_2_O_3_^2−^ + (x − 1) S^0^ + OH^−^ (3)

Sulfide addition was controlled via ORP, where an ORP value (ORP alarm) between −200 and −410 mV served as the set point. When the potential exceeded the ORP alarm threshold, the sulfide feed pump operated for a determined time (4–20 s). The system was monitored and controlled using LabVIEW ™ (National Instruments™, USA).

The bioreactor underwent a 35-day operational process, as detailed in Table 3.

Initially, activated sludge from a conventional wastewater treatment plant located in El Torno, Cádiz, Spain was used to inoculate the bioreactor (20%*v/v*), marking the commencement of the operation. This initial start-up phase, referred to as Stage I, extended for 13 days. During Stage I, the bioreactor operated with NO_2_^−^ serving as the primary electron acceptor. Nitrite was introduced into the bioreactor to achieve a target concentration of 14 g N-NO_2_^−^ L^−1^. At the end of this phase, a pH adjustment from 8.5 to 10 was implemented, favoring the growth of sulfur-oxidizing alkaliphilic microorganisms (Stage II). On the 20th day of operation, as the nitrite neared depletion, a key modification was introduced by substituting NO_2_^−^ with NO_3_^−^ as the primary electron acceptor at a concentration of 1 g N-NO_3_^−^ L^−1^ to investigate its impact on the process (Stage III). From day 24 onwards (Stage IV), the bioreactor was transitioned into batch mode until the end of the operation, with a particular focus on increasing thiosulfate concentration. On day 31 the air supply was intentionally cut, allowing for the assessment of its influence on thiosulfate production during Stage V. Bacterial growth was assessed through cell counting employing an enhanced Neubauer cell counting chamber (Marienfeld Superior™, Lauda-Königshofen, Germany) and an optical microscope (Olympus BH-2, Olympus Europa SE and Co. KG, Hamburg, Germany). Bioreactor samples were collected and subjected to suitable dilution. Triplicate measurements were performed to ensure the reliability and reproducibility of the results.

### 3.5. Effect of Lower Pulp Density and Biogenic Thiosulfate

In this phase, the ability of the thiosulfate-copper-ammonia system to leach Pd from a lower pulp density (5%*w/v*) of synthetic catalyst was studied. For this purpose, biogenic thiosulfate was used to test its leaching ability. The leaching tests were carried out following the same procedure stated in Section 2.2 with the difference of the pulp density (5% *w/v*) and the use of the optimized leaching solution with Na_2_S_2_O_3_ 1.2 M, CuSO_4_ 0.03 M, (NH_4_)_2_SO_4_ 1.5 M, Na_2_SO_3_ 0.2 M, pH 8, and 60 °C. To attain the target concentration of 1.2 M for biogenic thiosulfate, the liquid within the bioreactor (3 L) underwent a four-hour evaporation process at 100 °C. Following concentration and cooling, a significant portion of residual salts precipitated out. Subsequently, the liquid underwent filtration using a membrane filter with a pore size of 0.22 μm. This filtration step successfully eliminated these residual salts, resulting in a purified thiosulfate concentration within the culture medium.

### 3.6. Analytical Techniques

The metal content of the liquid and solid phases of the previous experiments was measured by induction-coupled plasma atomic emission spectroscopy (ICP-AES) of the UCA SC-ICYT (Iris Intrepid, Thermo Scientific, Waltham, MA, USA). Sulfate concentration was measured using the turbidimetric method (4500-SO_4_^2−^ E) [79]. Nitrite and nitrate were analyzed by a colorimetric method (4500-NO_2_^−^ B) and an ultraviolet spectrophotometric screening method (4500-NO_3_^−^ B), respectively [79], using a Spectroquant Pharo 300 spectrophotometer (Merck, Darmstadt, Germany). The thiosulfate concentration was measured by iodometric titration [80].

## 4. Conclusions

In conclusion, this study explored an innovative and environmentally friendly approach for the recovery of PGMs, focusing on the bioleaching of Pd from spent synthetic catalysts. The presented research introduces a novel method involving the utilization of a thiosulfate-copper-ammonia complex, with a unique twist—the incorporation of renewable biogenic thiosulfate obtained from a bioreactor engaged in the biodesulfurization of biogas. To achieve this, the production of biogenic thiosulfate was optimized by studying operational parameters such as pH, electron acceptors, and operation mode. Notably, a transition to the batch mode with aerobic conditions at pH 10 proved most effective for biogenic thiosulfate production. Under the most favorable conditions (batch mode, aerobic conditions, pH = 10, and an airflow rate of 0.033 vvm), a maximum concentration of 4.9 g L^−1^ of biogenic thiosulfate was obtained. However, the biomass growth resulting from the batch operation led to a subsequent decline in thiosulfate levels, indicating the potential utilization of thiosulfate as an energy source. The Pd bioleaching from the ground catalyst was subject to optimization, indicating the ideal conditions for Pd bioleaching to be Na_2_S_2_O_3_ 1.2 M, CuSO_4_ 0.03 M, (NH_4_)_2_SO_4_ 1.5 M, Na_2_SO_3_ 0.2 M, pH 8, and a temperature of 60 °C. The feasibility of using biogenic thiosulfate in the Pd bioleaching process was demonstrated achieving extraction rates of 93.2% of the total Pd from the solid. This underscores the viability of the proposed method as a sustainable and effective approach for Pd recovery from spent TWCs, addressing the growing demand for PGMs and contributing to the responsible utilization of critical raw materials in automotive catalysts. Future research should focus on a more detailed exploration of the impact of impurities in real spent automotive three-component catalysts on palladium leaching dynamics. The central aim of upcoming studies is to optimize the recovery of platinum group metals from automotive catalysts, achieving a balance among efficiency, environmental responsibility, and economic feasibility.

## Figures and Tables

**Table 1:** PB design for screening of significant factors affecting Pd leaching.

Run No.	Na_2_S_2_O_3_ (M)	CuSO_4_ (M)	(NH_4_)_2_SO_4_ (M)	Na_2_SO_3_ (M)	pH	T (°C)	Airflow Rate (vvm)	Pd Leaching (%)
1	1.2 (+)	0.06 (+)	1.5 (+)	0.0 (−)	12.0 (+)	60.0 (+)	0 (−)	2.0
2	0.6 (−)	0.06 (+)	1.5 (+)	0.0 (−)	12.0 (+)	25.0 (−)	0 (−)	1.1
3	1.2 (+)	0.03 (−)	0.5 (−)	0.0 (−)	12.0 (+)	60.0 (+)	2 (+)	1.0
4	0.6 (−)	0.03 (−)	0.5 (−)	0.1 (+)	12.0 (+)	60.0 (+)	0 (−)	1.4
5	0.6 (−)	0.06 (+)	0.5 (−)	0.0 (−)	8.0 (−)	60.0 (+)	2 (+)	0.1
6	1.2 (+)	0.03 (−)	1.5 (+)	0.0 (−)	8.0 (−)	25.0 (−)	2 (+)	0.1
7	1.2 (+)	0.03 (−)	1.5 (+)	0.1 (+)	8.0 (−)	60.0 (+)	0 (−)	26.0
8	1.2 (+)	0.06 (+)	0.5 (−)	0.1 (+)	12.0 (+)	25.0 (−)	2 (+)	0.6
9	0.6 (−)	0.03 (−)	1.5 (+)	0.1 (+)	12.0 (+)	25.0 (−)	2 (+)	1.0
10	1.2 (+)	0.06 (+)	0.5 (−)	0.1 (+)	8.0 (−)	25.0 (−)	0 (−)	0.1
11	0.6 (−)	0.06 (+)	1.5 (+)	0.1 (+)	8.0 (−)	60.0 (+)	2 (+)	0.1
12	0.6 (−)	0.03 (−)	0.5 (−)	0.0 (−)	8.0 (−)	25.0 (−)	0 (−)	0.1

**Table 2:** Experimental design for analyzing the impact of each variable.

Test	Na_2_S_2_O_3_ (M)	CuSO_4_ (M)	(NH_4_)_2_SO_4_ (M)	Na_2_SO_3_ (M)	pH
A	2.4	0.030	1.5	0.1	8.0
B	1.2	0.015	1.5	0.1	8.0
C	1.2	0.030	3.0	0.1	8.0
D	1.2	0.030	1.5	0.2	8.0
E	1.2	0.030	1.5	0.1	6.0

**Table 3:** Operating parameters of the gas-lift bioreactor.

Stage	Time (days)	Action	pH	O_2_ (vvm)	Electron Acceptor Source	Operation Mode	HRT (Days)
I	0–13	Start-up	8.5	0.033	NO_2_^−^	Continuous	3.5
II	13–20	pH increase	10	0.033	NO_2_^−^	Continuous	3.5
III	20–24	Anoxic operation (nitrate)	10	0.033	NO_3_^−^	Continuous	3.5
IV	24–31	Batch mode	10	0.033	NO_3_^−^	Batch	-
V	31–35	Air supply cut	10	0	NO_3_^−^	Batch	-

## Data Availability

Data will be made available on request.

## Supplementary Materials

The following supporting information can be downloaded at: https://www.mdpi.com/article/10.3390/molecules28248078/s1, Figure S1: Standardized effect size of the studied factors on Pd leaching.; Figure S2: Precipitation of Pd after separation of the liquid and the solid phase through filtration with 0.22 µm nitrocellulose membranes (a) in the solid phase and (b) in the liquid phase.
