# Supplementary figures and images for: Sustainable Recovery of Platinum Group Metals from Spent Automotive Three-Way Catalysts through a Biogenic Thiosulfate-Copper-Ammonia System
Source: Molecules. 2023 Dec 14;28(24):8078. doi: 10.3390/molecules28248078 (PMC10746061; doi:10.3390/molecules28248078)

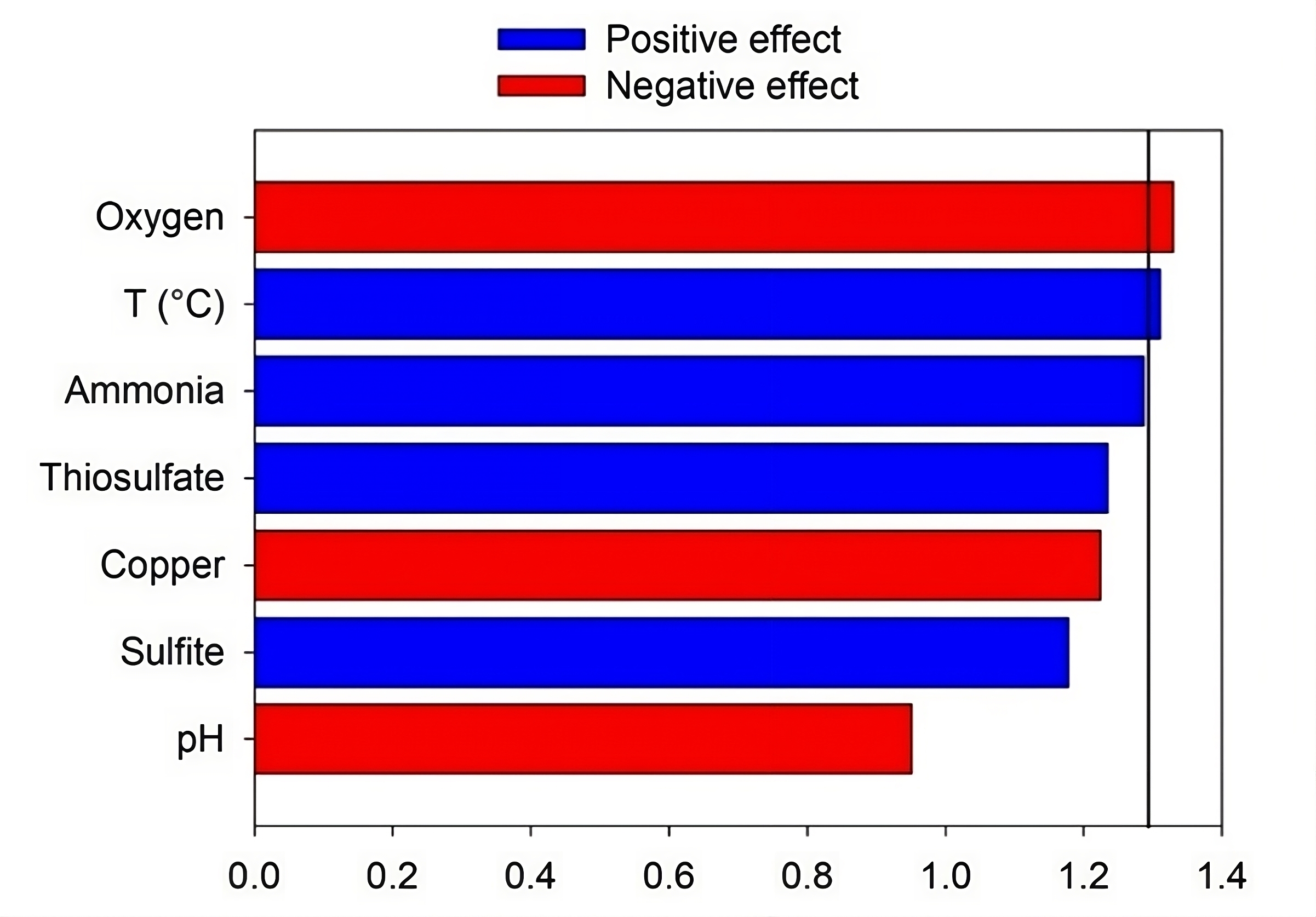

Supplement: Supplementary file 1 [file molecules-28-08078-s001.zip › Fig. S1.jpg]

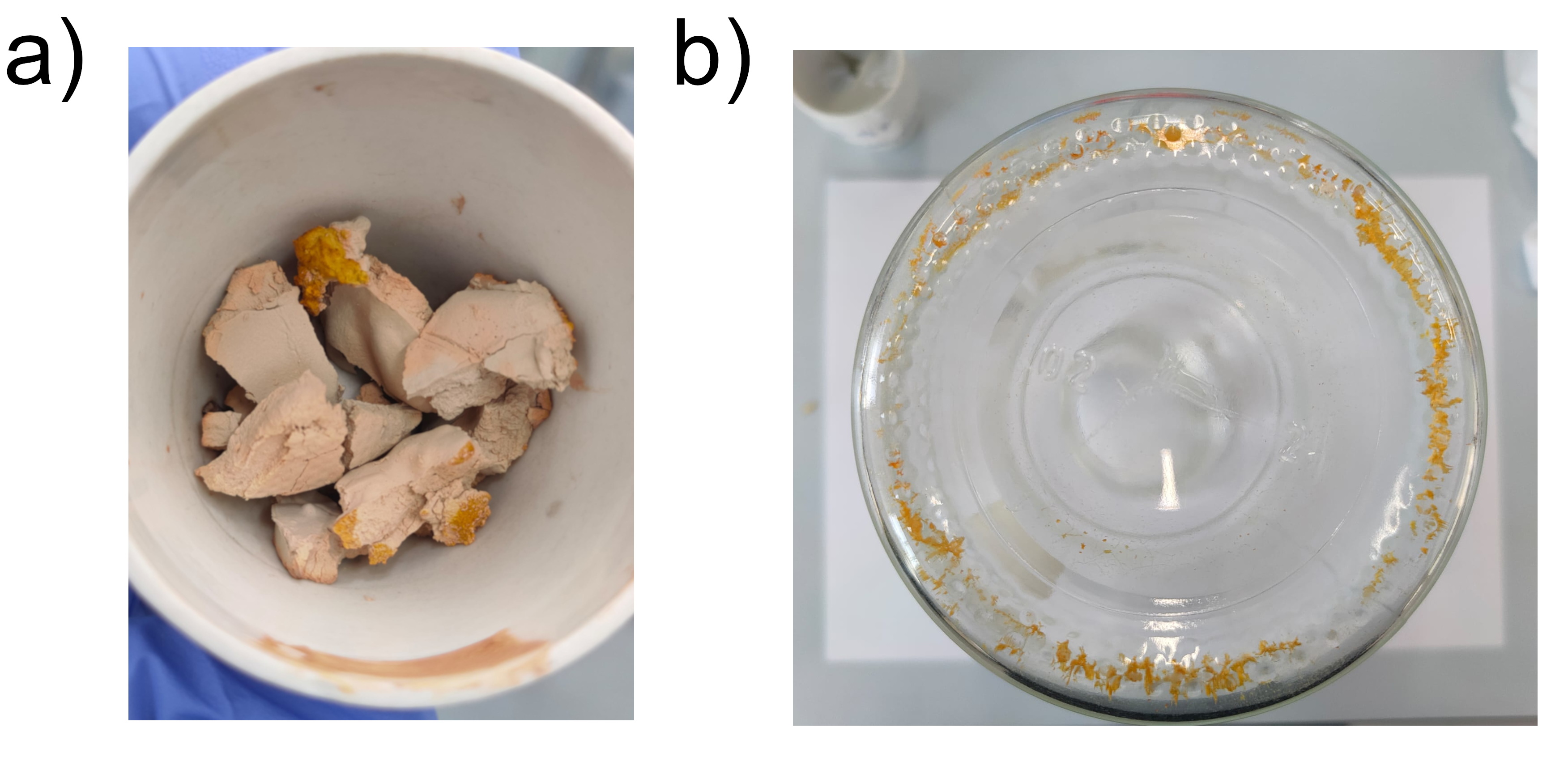

Supplement: Supplementary file 1 [file molecules-28-08078-s001.zip › Fig. S2.png]
